# Differential Functions of Two Metalloproteases, *Mrmep1* and *Mrmep2*, in Growth, Sporulation, Cell Wall Integrity, and Virulence in the Filamentous Fungus *Metarhizium robertsii*

**DOI:** 10.3389/fmicb.2018.01528

**Published:** 2018-07-06

**Authors:** Rong Zhou, Xiazhi Zhou, Ali Fan, Zhangxun Wang, Bo Huang

**Affiliations:** ^1^Anhui Provincial Key Laboratory of Microbial Pest Control, Anhui Agricultural University, Hefei, China; ^2^School of Plant Protection, Anhui Agricultural University, Hefei, China

**Keywords:** *Metarhizium*, metalloprotease, virulence, sporulation, cell wall integrity

## Abstract

The *Metarhizium* genus of filamentous entomopathogenic fungi plays a pivotal role in regulating insect populations. Metalloproteases (MEPs) are a widely distributed and diverse family of hydrolytic enzymes that are important toxicity factors in the interactions between fungi and their hosts. Herein, we characterized two MEPs, Mrmep1 and Mrmep2, in *Metarhizium robertsii* using gene deletion. Growth rates of the resulting Δ*Mrmep1* and Δ*Mrmep2* mutants decreased by 16.2 and 16.5%, respectively, relative to the wild-type (WT) strain. Both mutants were less sensitive to cell wall-perturbing agents, sodium dodecyl sulfate and Congo red than the WT strain, whereas did not show any obvious changes in fungal sensitivity to ultraviolet B irradiation or heat stress. The conidial yield of Δ*Mrmep1*, Δ*Mrmep2*, and Δ*Mrmep1*Δ*Mrmep2* mutants decreased by 56.0, 23, and 53%, respectively. Insect bioassay revealed that median lethal time values against *Galleria mellonella* increased by 25.5% (Δ*Mrmep1*), 19% (Δ*Mrmep2*), and 28.8% (Δ*Mrmep1*Δ*Mrmep2*) compared with the WT strain at a concentration of 1 × 10^7^ conidia mL^-1^, suggesting attenuated fungal virulence in the Δ*Mrmep1*, Δ*Mrmep2,* and Δ*Mrmep1*Δ*Mrmep2* strains. During fungal infection, transcription levels of *Mrmep1* was 1.6-fold higher than *Mrmep2* at 36 h post inoculation. Additionally, transcription levels of gallerimycin gene were 1.2-fold, 2.18-fold, and 2.5-fold higher in insects infected with the Δ*Mrmep1*, Δ*Mrmep2,* or Δ*Mrmep1*Δ*Mrmep2* mutant than those infected with the WT strain, respectively. Our findings suggest that *Mrmep1* and *Mrmep2* are differentially contributed to the growth, sporulation, cell wall integrity, and virulence of *M. robertsii*.

## Introduction

*Metarhizium robertsii* is an entomopathogenic fungus that is widely used in the biological control of a variety of insects that cause significant economic losses in agriculture (Frazzon et al., 2000; Lord, 2005; Faria and Wraight, 2007). *Metarhizium* spp. utilize their abundant hydrolytic enzymes, including chitinases, proteases, lipases, and esterases, to penetrate insect cuticles, which consist of proteins, chitins, and lipids (Adams, 2003; Leger and Wang, 2010; Wang and Feng, 2014). Proteases from pathogenic fungi not only degrade the insect body wall but also activate the insect immune system (Gillespie et al., 2000; Fernandes et al., 2012). Of these proteins, metalloproteinases are a type of protease that rely on metal ions for activation (Tallant et al., 2006). Zinc metalloproteases (MEPs) depend on zinc ions and have a small number of common HEXXH sequences. Depending on the nature of the protein and the position of the third ligand in the zinc ion, zinc MEPs are divided into three groups, metzincins, aspzincins, and gluzincins (Laursen et al., 2002; Glerup et al., 2005; Tallant et al., 2006).

Metalloproteases of pathogens have been linked to virulence (Zhang et al., 1999; Dow et al., 2000; Jia et al., 2000). In the rice blast fungus *Magnaporthe oryzae*, the zinc MEPs Avr-Pita triggers a signaling transduction cascade recognized by a cytoplasmic receptor Pita (Jia et al., 2000). The secreted fungalysin MEPs gene *cgfl* is strongly upregulated during the early stages of infection in *Colletotrichum graminicola*, suggesting a role in fungus-plant interaction (Vargas et al., 2012; Sanzmartín et al., 2015). ZrMEP1, the first reported MEPs from an entomopathogenic fungus *Zoophthora radicans*, is associated with pathogenesis but not a major host specific determinant (Xu et al., 2006).

The role of entomopathogenic fungal MEPs in growth, germination, stress tolerance, and virulence has not yet been characterized. In our previous study, two MEP genes, *Mrmep1* (EFY97549) and *Mrmep2* (EFY97706), were significantly upregulated in heat-treated conidia (Wang et al., 2014). Here, we characterized the two MEPs by generating gene-disruption mutants. Our results demonstrated that the two MEPs are involved in growth, sporulation, cell wall integrity, and virulence, but their contributions to fungal virulence are different.

## Materials and Methods

### Fungal Strains, Host Insects, and Culture Conditions

The fungus *M. robertsii* strain ARSEF 23 was kindly gifted by Dr. Chengshu Wang (Gao et al., 2011). The fungal strains were cultured on potato dextrose agar [PDA, 20% potato, 2% dextrose and 2% agar (w/v)] medium at 25°C for 14 days in the dark. Collected conidia were dispersed in sterile 0.05% Tween-80 solution and filtered through non-woven fabric to remove mycelia. The conidial suspension was inoculated into Sabouraud dextrose agar yeast extract culture medium (SDAY; 4% dextrose, 1% peptone, 1% yeast extract, and 1.5% agar) and incubated at 25°C for 3 days and hyphae/cultures were harvested by scraping from the cellophane. *Escherichia coli* DH5α were cultured at 37°C in Luria Bertani broth (LB; 1% tryptone, 0.5% yeast extract, and 1% NaCl [w/v]). The *Agrobacterium tumefaciens* AGL-1 stain containing target plasmid used as a T-DNA donor for fungal transformation and was incubated in yeast extract beef broth (YEB; 0.5% sucrose, 0.1% yeast extract, 1% peptone, and 0.05% MgSO_4_⋅7H_2_O) at 28°C for 16–20 h. For fungal virulence bioassay, larvae of the great wax moth *Galleria mellonella* were obtained from RuiQing Bait Co., Ltd. (Shanghai, China) and used for the bioassay.

### Cloning, Bioinformatics, and Phylogenetic Analysis of MEPs

The sequences of genes encoding MrMEP1 (EFY97549) and MrMEP2 (EFY97706) were obtained from the NCBI database. Primers were designed to amplify the complete cDNA, including the 5′ untranslated region (UTR) and 3′ UTR, using a SMART RACE cDNA Amplification Kit (Clontech, Mountain View, CA, United States after which the products were cloned and sequenced. Domain analysis was performed using the conserved domain database (CDD^1^). Protein parameters were calculated using the ProtParam tool in ExPASy^2^ and signal peptide prediction was carried out using the SignalP 4.0 server^3^.

Homologous MEP sequences from different fungal species were retrieved from the NCBI database^4^. For phylogenetic analysis, MEP sequences were aligned using ClustalX and then a neighbor-joining (NJ) tree was generated using MEGA 7.0 software (Wang et al., 2017).

### Generation of Gene Deletion and Complementation Mutants

Gene deletion and complementation tests were conducted using a method described previously (Fang et al., 2006). All primers are listed in **Table 1**.

**Table 1:** Sequences of primers used.

Gene	Primer name	5′to 3′ sequence	Notes
**Gene deletion**		
*Mrmep1*	*Mrmep1*-5F	AACTGCAGTAGCAAGCATAGCCAGTC	PCR identification of *Mrmep1*
	*Mrmep1-*5R	AACTGCAGAGTGGCCTTTACTTTGAG	Deletion transformants
	*Mrmep1*-3F	GGACTAGTTCGATTTAGTGGGAATGAGC	
	*Mrmep1*-3R	GCTCTAGACATCGCTTGAATCTCCTGTG	
	*Mrmep1*-F	ACAACCAGATGGACGTGCTAAAC	
	*Mrmep1*-R	TAACTGTTGGCAAAGTCCACCTC	
*Mrmep2*	*Mrmep2*-5F	AACTGCAGCGAGTTTGGGAAAGTAGTGC	PCR identification of MrMEP2
	*Mrmep2*-5R	AACTGCAGCAGACGGCTGGTTGTAAGA	Deletion transformants
	*Mrmep2*-3F	GACTAGTGTCTTCCGATGGCTGCTT	
	*Mrmep2*-3R	GCTCTAGACATGCCTTGGTTGTCTGC	
	*Mrmep2*-F	AGGCACTCCAACCAATTAGCA	
	*Mrmep2*-R	AAGCAGCCATCGGAAGACAAGG	
*bar*	*bar*-F	TCGTCAACCACTACATCGAGAC	
	*bar*-R	GAAGTCCAGCTGCCAGAAAAC	
*ben*	*ben*-F	GGTAACTCCACCGCCATCCA	
	*ben*-R	GCAGGGTATTGCCTTTGGACTT	
**Gene complementation**		
*Mrmep1*	c*Mrmep1*-5F	CACGCCTGTCGTATTCCAGCATT	
	c*Mrmep1*-3R	TGTTCTTGATGTTGTGCTCGCCC	
*Mrmep2*	c*Mrmep2*-5F	CCGCTCGAGCGCCGCTCTTTACGTTCTTT	
	c*Mrmep2*-3R	AACTGCAGTTACTCGTAGGAATACCCCATG	
Southern blotting			
	S-*bar*	GCCGTGCCACCGAGGCGGACATGCCGGCGGTCTGCACCATCGTCAACCACTACATCGAG	
	S-*ben*	CCCCTTCTGTGCCTCTACCTACTGCTGCCCGACTCATTATGATCCTGCTCGCTTTCTCG	
**Gene expression analysis and RT-PCR identification of deletion transformants**		
*Mrmep1*	*Mrmep1*-FF	GGCTTGCACCCCATTATCACC	
	*Mrmep1*-RR	CCATTGCTTGTCTGCCTCTGTTT	
*Mrmep2*	*Mrmep2*-FF	TGATGGAGAAGCGGCAAAATG	
	*Mrmep2*-RR	GGCATCACCCCTGTGGTATCTT	
Gallerimycin	*gal*-F	CTACAGAATCACACGACACT	
	*gal*-R	CGAAGACATTGACATCCATT	
*gpd*	*gpd*-F	GACTGCCCGCATTGAGAAG	
	*gpd* -R	AGATGGAGGAGTTGGTGTTG	
18S	18S-F	CGCGCTACACTGAAGGAATC	
	18S-R	TTGATTACGTCCCTGCCCTT	

For gene deletion, fragments of the 5′- and 3′-end-flanking regions of *Mrmep1* (1200 and 1000 bp, respectively) were amplified using genomic DNA as the PCR template and Dream Taq DNA Polymerase (Fermentas, Burlington, ON, Canada) with the primer pairs *Mrmep1*-5F/*Mrmep1*-5R and *Mrmep1*-3F/*Mrmep1*-3R. The purified 5′- and 3′-end-flanking fragments were subsequently cloned into the *Pst*I and *Spe*I/*Xba*I restriction enzyme sites in the binary vector pDHt-SK-bar (conferring resistance against ammonium glufosinate) to produce the binary vector p*bar*-*Mrmep1* for *Agrobacterium*-mediated fungal transformation where a 1993-bp fragment was replaced by the bar cassette. The *Mrmep2* deletion mutant was constructed as described above but with the binary vector pDHt-SK-ben. The length of the 5′- and 3′-end flanking regions as well as the replaced fragment in the Δ*Mrmep2* mutant were 641, 550, and 581 bp, respectively.

For gene complementation, the complementation vector C-p*ben*-*Mrmep1* was constructed by inserting the entire *Mrmep1* gene plus 1000 bp of the upstream sequence and 200 bp of the downstream sequence into the binary vector pDHt-SK-ben for fungal transformation. Construction of the *Mrmep2* complementation vector and fungal transformation were the same as that for *Mrmep1*, except that the binary vector pDHt-SK-bar was used instead.

For double gene disruption, *Mrmep2* was disrupted in a Δ*Mrmep1* background. For confirmation analysis, all mutants were verified by PCR and reverse transcription PCR (RT-PCR) (**Table 1**). For Δ*Mrmep1* deletion and complementation strains, PCR using genomic DNA and two primer sets (*Mrmep1*-F *and Mrmep1*-R; *Mrmep1*-F and *bar*-R) was performed for detecting the two fragments of interest (1000-bp partial *Mrmep1* fragment and 2000-bp partial *Mrmep1* plus partial *bar* fragment) in different strains; the *bar* gene was also detected using the primer set *bar*-F and *bar*-R in the same strains. RT-PCR analysis was conducted to detect the present partial *Mrmep*1 fragment in Δ*Mrmep1* using cDNA as the template; the glyceraldehyde 3-phosphate dehydrogenase (*gpd*) gene (MAA_07675) was used as an internal control. For the other single and double mutants, the same method as described above was used but with different primer sets.

For Southern blotting, 30 μg samples of genomic DNA extracted from the SDAY colonies of wild-type (WT) and each mutant were digested with *BamH*I and *Pst*I. After separation on 0.7% agarose gels, the DNA fragments were transferred to a nitrocellulose membrane and probed with two *bar* and *ben* gene fragments labeled with the PCR DIG Labeling Mix Kit (Roche, Basel, Switzerland. Probe preparation, membrane hybridization, and visualization were performed according to manufacturer’s instructions (DIG High Prime DNA Labeling and Detection Starter Kit II; Roche).

### Mutant Phenotype Assays

Phenotype assays were performed as described previously (Meng et al., 2017). The conidial suspension (1 μL; concentration, 1 × 10^7^ conidia mL^-1^) of each strain was spotted onto various media unless otherwise noted.

For the growth assay, conidial suspensions of WT and mutant strains were spotted in the center of PDA plates and radial growth (colony diameter) of the vegetative mycelia at 25°C was measured daily.

For the sporulation assay, 30 μL conidia suspension at a concentration of 1 × 10^7^ conidia mL^-1^ was spread onto PDA plates and incubated at 25°C for 14 days to evaluate sporulation capacity.

For chemical stress tolerance assays, conidial suspensions of WT and mutants were spotted in the center of PDA plates containing different chemical reagents, including the cell wall-disturbingcompounds Congo red (3 mg mL^-1^) or sodium dodecyl sulfate (SDS)(2.5 μg mL^-1^; Ying and Feng, 2011). PDA plates without reagents were used as controls. The rate of growth inhibition (RGI%) was calculated as (C - S)/C × 100, where C is the growth rate of the control and S is the growth rate under stress conditions (Wang et al., 2017).

For heat stress tolerance assays, we placed 1-mL conidial suspension aliquots of WT and each mutant strain in 1.5-mL Eppendorf tubes and incubated in a water bath at 40°C for 90 min. One hundred microliter conidial suspension from each tube were inoculated on PDA plates. Conidial germination was observed under the microscope after 24 h of incubation. Conidia with visible germ tubes were considered germinated.

To examine ultraviolet B (UV-B) stress tolerance, 30-μL aliquots of conidial suspensions were centrally smeared onto petri dishes and exposed to UV-B irradiation with a wavelength of 312 nm (280–320 nm) at 100 μJ cm^-2^ using a UV crosslinker (HL-2000 Hybrilinker; UVP, Upland, CA, United States) (Yao et al., 2010). After exposure, conidial germination on each plate was observed as described for the heat stress tolerance assay.

Bioassays were conducted using *G. mellonella* larvae as described previously (Wang et al., 2017). Insects were immerged in conidial suspensions for 90 s. All immersed larvae were maintained in large Petri dishes for 10 days at 25°C and examined every 12 h for mortality records. The SPSS statistical package was used to determine the median lethal time (LT_50_) values. All bioassays were repeated three times in triplicate with 15 insects per replicate.

### Quantitative RT PCR (qRT-PCR)

First strand cDNA, to be used as the qPCR template, was synthesized from 1 μg total RNA extracted as described previously (Meng et al., 2017) with reverse transcriptase (TaKaRa, Dalian, China). qRT-PCR was carried out on a Real-Time PCR System (CFX Manager Software; Bio-Rad, Hercules, CA, United States) using a SYBR Green kit (SYBR Premix Ex Taq II; TaKaRa Dalian, China) according to manufacturer’s instructions. Amplification conditions for qPCR were 95°C for 5 s, followed by 40 cycles of 95°C for 5 s and 60°C for 30 s; all reactions were run in triplicate. The threshold cycle (Ct) was determined using default threshold settings. We used the ΔΔCt method to calculate relative gene expression levels (Livak and Schmittgen, 2001) using *gpd* (GenBank accession number MAA_07675) as an internal control for each sample (Fang and Bidochka, 2006).

To investigate the expression patterns of *Mrmep1* and *Mrmep2* during infection, total RNA was extracted from mixed fungi and insect samples by TRIzol reagent (Invitrogen, Camarillo, CA, United States) and used for qRT-PCR q(with *gpd* as internal control) at different time points of 6, 12, 24, 36, and 48 h after inoculation. The gallerimycin gene is an antifungal peptide in the Lepidoptera insect that can be induced by a variety of bacteria, fungi, and even MEPs in the corpus adiposum. This peptide plays an important role in insect humoral immunity (Feng et al., 2015; Cen et al., 2017). Therefore, we chose the 36 h post inoculation (hpi) time point to examine how the insect responds to the fungus, relative to the 6 h expression levels of the host gallerimycin gene. Additionally, 18S rRNA (GenBank accession number AF286298) was used as the reference in qRT-PCR.

### Statistical Analysis

All data were analyzed using GraphPad Prism 5 (GraphPad Software, La Jolla, CA, United States). Data were expressed as the mean ± standard error (SE) of at least three independent experiments. Student’s *t*-test was used to compare the differences between two means. For multiple comparisons, Tukey’s multiple comparison test was used for significance analysis. *p*-values equal to or less than 0.05 were considered significant.

## Results

### Characteristics of *Mrmep1* and *Mrmep2* in *M. robertsii*

The two MEP sequences were amplified using rapid-amplification of cDNA ends (RACE). *Mrmep1* (EFY97549) has a 93-bp 5′ UTR and a 105-bp 3′ UTR and encodes a polypeptide of 611 amino acids. *Mrmep2* (EFY97706) includes 5′ and 3′ UTRs of 74 and 101 bp, respectively, and encodes a 255-amino acid polypeptide.

The predicted molecular weight and theoretical isoelectric point (pI) of the deduced MrMEP1 and MrMEP2 proteins were 68.66 and 27.89 kDa, and 6.17 and 5.66, respectively. Both MrMEP1 and MrMEP2 were found to contain predicted signal peptide sequences, suggesting that they are extracellular proteins.

**FIGURE 1:**
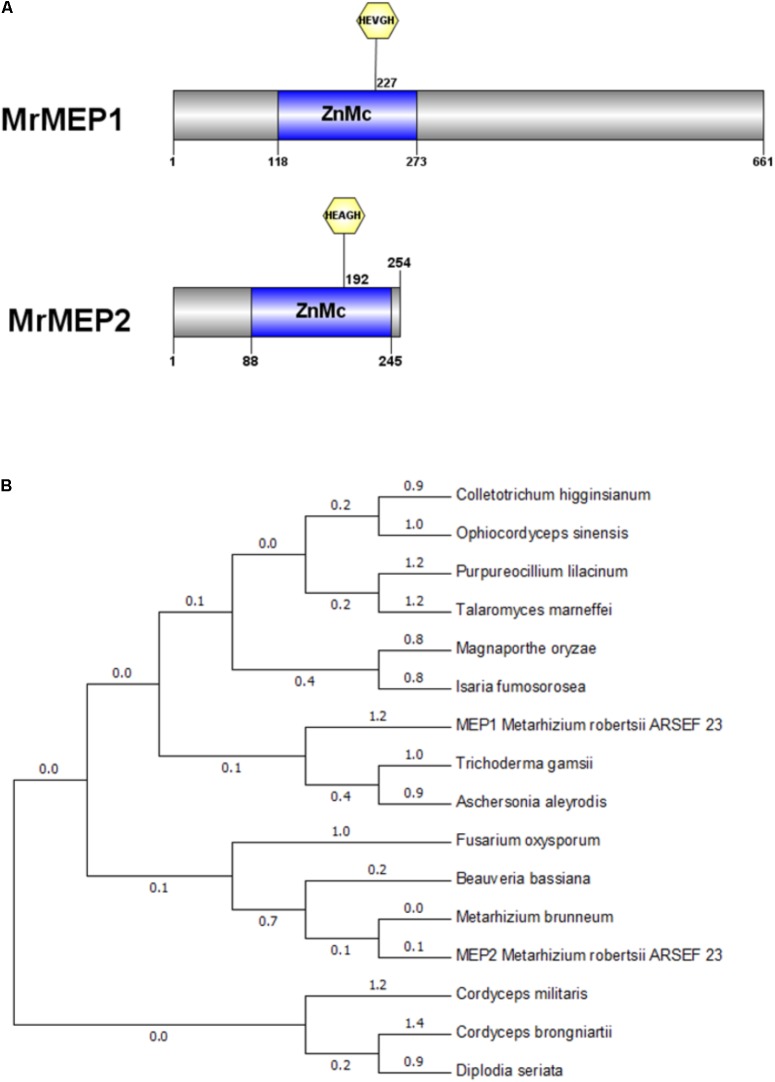
Bioinformatic analysis of MrMEP1 and MrMEP2 proteins. **(A)** The structural domain sites in MrMEP1 and MrMEP2 proteins. **(B)** Phylogenetic analysis of the fungal MEP proteins. Phylogenetic trees constructed with fungal MEP proteins. The protein accession numbers as follows, EWZ30531.1 hypothetical protein FOZG_16010 [*Fusarium oxysporum* Fo47]; PNP41669.1 hypothetical protein TGAMA5MH_06456 [*Trichoderma gamsii*]; XP_018155334.1 metalloprotease [*Colletotrichum higginsianum* IMI 349063]; XP_018179452.1 metalloprotease [*Purpureocillium lilacinum*]; OAA39196.1 metalloprotease 1 [*Cordyceps brongniartii* RCEF 3172]; XP_003714993.1 metalloprotease 1 [*Magnaporthe oryzae* 70-15]; XP_014540809.1 metalloprotease 1, partial [*Metarhizium brunneum* ARSEF 3297]; KZZ90284.1 metalloprotease MEP1 [*Aschersonia aleyrodis* RCEF 2490]; XP_008593388.1 metalloprotease MEP1 [*Beauveria bassiana* ARSEF 2860]; XP_002149130.1 metalloprotease MEP1 [*Talaromyces marneffei* ATCC 18224]; XP_002149130.1 metalloprotease MEP1 [*Talaromyces marneffei* ATCC 18224]; XP_018708221.1 peptidase M43, pregnancy-associated plasma-A [*Isaria fumosorosea* ARSEF 2679]; XP_006674200.1 peptidase M43B, pregnancy-associated plasma-A [*Cordyceps militaris* CM01]; EQL01370.1 protein related to metalloprotease MEP1 [*Ophiocordyceps sinensis* CO18]; KKY13372.1 putative metalloprotease 1 [*Diplodia seriata*]; EFY97549.1 metalloprotease MEP1-like protein [*Metarhizium robertsii* ARSEF 23]; EFY97706.1 metalloprotease 1 [*Metarhizium robertsii* ARSEF 23].

Bioinformatics analysis indicated that a ZnMC domain was present at residues spanning 118-273 in MrMEP1 and 88-245 in MrMEP2 (**Figure 1A**). Furthermore, the active site motifs **HE**VG**H**WLGLL**H**PHE (MrMEP1), **HE**AG**H**WLGLL**H**TFE (MrMEP2), the Met-Turn motifs HNY**M**TY (MrMEP1) and HNYMGY (MrMEP2) were identified by analyzing the amino acid sequences at the domain sites of MrMEP1 and MrMEP2. These data confirmed that both proteins are Zn^2+^ MEPs.

A phylogenetic tree of MEPs from *Metarhizium* spp. and related fungal species was constructed using *Cordyceps militaris* as the outgroup, revealing that MrMEP1 is most closely related to *Trichoderma gamsii* and *Isaria fumosorosea* MEPs, whereas MrMEP2 is more closely related to *Metarhizium brunneum* MEPs (**Figure 1B**).

### Knockout and Complementation of *Mrmep1* and *Mrmep2*

To investigate the roles of these two MEPs in *M. robertsii*, five gene replacement or complementation strains were generated, which include Δ*Mrmep1* (*Mrmep1* disruption mutant), Δ*Mrmep2* (*Mrmep2* disruption mutant), cpΔ*Mrmep1* (*Mrmep1* complementation strain), cpΔ*Mrmep2* (*Mrmep2* complementation strain), and Δ*Mrmep1*Δ*Mrmep2* (*Mrmep1* and *Mrmep2* double disruption mutant) (**Supplementary Figure S1**). All mutant strains were confirmed by PCR using genomic DNA or cDNA as a template.

**FIGURE 2:**
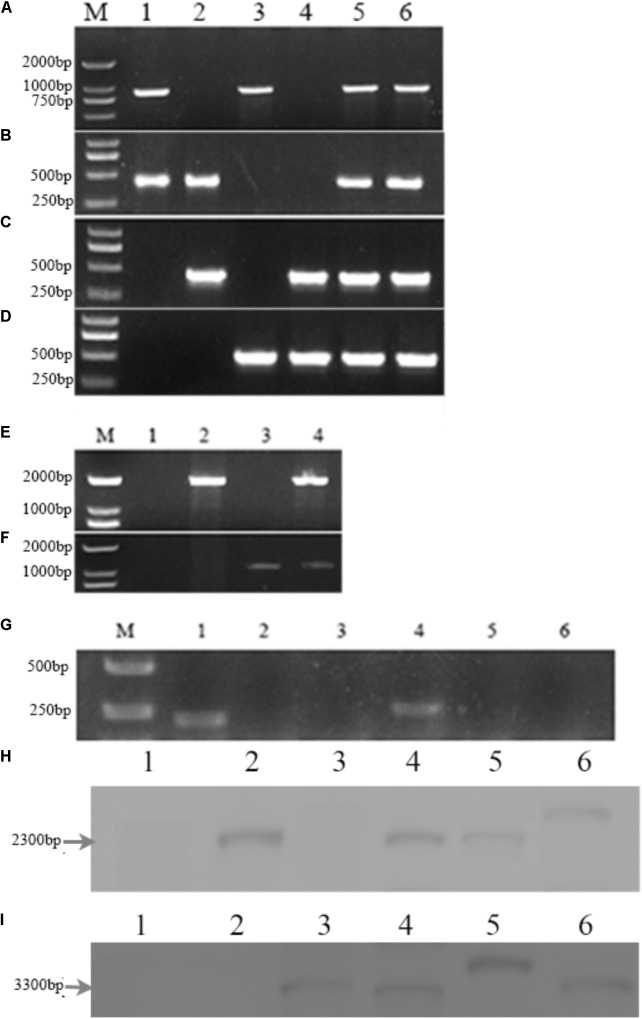
Deletion of the *Mrmep1* and *Mrmep2* gene in *M. robertsii*. **(A–D)** PCR analysis of the deleted genes using the primers *Mrmep1*-F and *Mrmep1*-R **(A)**, the primers *Mrmep2*-F and *Mrmep2*-R **(B)**, the primers *bar*-F and *bar*-R **(C)**, and the primers *ben*-F and *ben*-R **(D)**. M, Marker; 1, WT; 2, Δ*Mrmep1*; 3, Δ*Mrmep2*; 4, Δ*Mrmep1* Δ*Mrmep2*; 5, cpΔ*Mrmep1*; 6, cpΔ*Mrmep2*. **(E,F)** PCR analysis for the *bar* **(E)** or *ben* **(F)** genes. M, Marker; 1, WT; 2, Δ*Mrmep1*; 3, Δ*Mrmep2*; 4, Δ*Mrmep1*Δ*Mrmep2*. **(G)** RT-PCR analysis for the deleted genes. M, Marker; 1 and 4, WT; 2, Δ*Mrmep1*; 3 and 6, Δ*Mrmep1*Δ*Mrmep2*; 5, Δ*Mrmep2*. **(H,I)** Southern blotting hybridization with *bar* or *ben* gene probes. 1, WT; 2, Δ*Mrmep1*; 3, Δ*Mrmep2*; 4, Δ*Mrmep1*Δ*Mrmep2*; 5, cpΔ*Mrmep1*; 6, cpΔ*Mrmep2*. Further information on primers can be found in **Table 1**.

PCR analysis showed that an 853-bp *Mrmep1* fragment was present in WT and cpΔ*Mrmep1,* but not in the Δ*Mrmep1* and Δ*Mrmep1*Δ*Mrmep2* strains (**Figure 2A**). In addition, a 413-bp *Mrmep2* fragment was detected in WT and cpΔ*Mrmep2,* but not in the Δ*Mrmep2* and Δ*Mrmep1*Δ*Mrmep2* strains (**Figure 2B**). A partial fragment (434 bp) corresponding to the *bar* gene was present in Δ*Mrmep1* and Δ*Mrmep1*Δ*Mrmep2* (**Figure 2C**) and a partial fragment (301 bp) corresponding to the *ben* gene was present in Δ*Mrmep2* and Δ*Mrmep1*Δ*Mrmep2* (**Figure 2D**). Furthermore, PCR analysis showed that a 2084-bp fragment and a 1104-bp fragment were detected by the primer sets *Mrmep1*-F/*bar*-R and *Mrmep2*-F/*ben*-R in the Δ*Mrmep1* and Δ*Mrmep2* strains, respectively (**Figures 2E,F**). Moreover, RT-PCR analysis confirmed loss or regain of the target gene transcription in the gene disruption mutants and reverse complement strains (**Figure 2G**).

Southern blotting further revealed the single copy insertion events in the mutants. The *bar* gene fragment probe detected a 2.3-kb *Bam*HI band in the Δ*Mrmep1*, Δ*Mrmep1*Δ*Mrmep2*, cpΔ*Mrmep1*, and cpΔ*Mrmep2* strains, but not in the WT and Δ*Mrmep2* strains (**Figure 2H**). The *ben* gene fragment probe detected a 3.3-kb *Bam*HI band in the Δ*Mrmep2*, Δ*Mrmep1*Δ*Mrmep2*, cpΔ*Mrmep1*, and cpΔ*Mrmep2* strains, but not in the WT and Δ*Mrmep1* strains (**Figure 2I**).

### *Mrmep1* and *Mrmep2* Are Involved in Growth and Development

**FIGURE 3:**
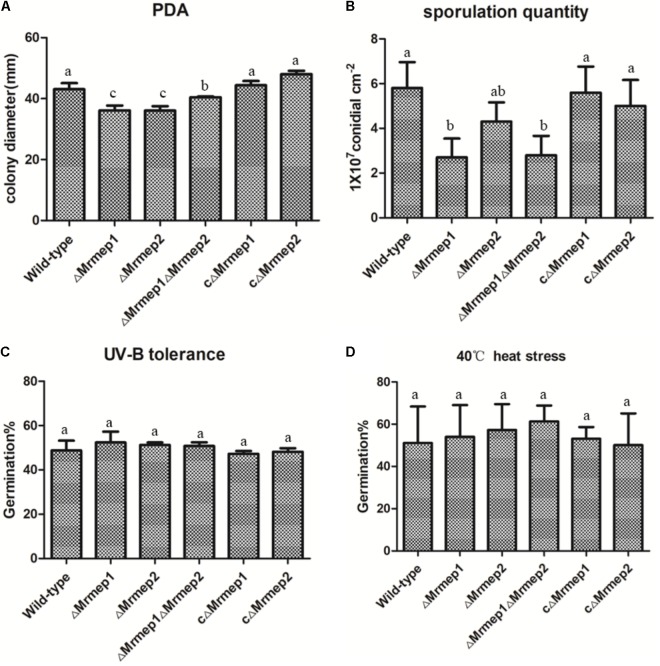
Wild-type (WT) and mutant strain growth rates and germination in PDA as well as heat and UV-B stress tolerance. **(A)** WT and mutant strains were spot inoculated onto PDA plates. **(B)** Sporulation quantity of the strains at 14 days. **(C)** WT and mutant strains were cultivated at 40°C under heat stress for 60 min. **(D)** WT and mutant strains were cultivated under UV-B irradiation for 90 s.

The effect on vegetative growth due to each MEP mutation was investigated. The growth rate of the Δ*Mrmep1* and Δ*Mrmep2* mutants were markedly reduced with a 16.2% (*p* < 0.05) and 16.5% (*p* < 0.05) decrease as compared to the WT strain, respectively (**Figure 3A**).

We next assessed the effect of disrupting MEPs on conidial yield. The results indicated that conidial yield in the Δ*Mrmep1*, Δ*Mrmep2*, and Δ*Mrmep1*Δ*Mrmep2* mutants was reduced by 56.0, 23, and 53%, respectively, compared with the WT. The Δ*Mrmep1* and Δ*Mrmep1*Δ*Mrmep2* mutants exhibited a considerably larger decrease, suggesting that *Mrmep1* has a more significant role than *Mrmep2* in conidiation (**Figure 3B**).

### *Mrmep1* and *Mrmep2* Are Involved in Cell Wall Integrity

**FIGURE 4:**
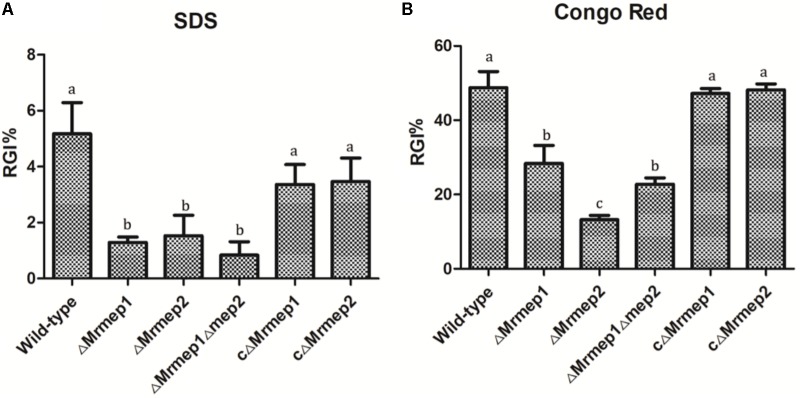
The rate of growth inhibition (RGI%) of the mutants in cell wall-disrupting compounds. **(A)** Mutant strains were spot inoculated onto PDA plates with 0.025% SDS. **(B)** Mutant strains were spot inoculated onto PDA plates with 0.3 mg mL^-1^ Congo red.

To examine the role of *Mrmep1* and *Mrmep2* in cell wall integrity, we measured mycelial growth on PDA containing SDS and CR. The SDS RGI values for WT, Δ*Mrmep1*, Δ*Mrmep2*, and Δ*Mrmep1*Δ*Mrmep2* were 5.67, 1.53, 1.29, and 0.83%, respectively. Meanwhile, the CR RGI values for WT, Δ*Mrmep1*, Δ*Mrmep2*, and Δ*Mrmep1*Δ*Mrmep2* were 48.79, 13.24, 28.38, and 22.78%, respectively. Thus, the sensitivity of the mutants to SDS and CR decreased significantly compared to that of the WT strain, suggesting roles of Mrmep1 and Mrmep2 in cell wall integrity (**Figure 4**).

To investigate whether the mutants exhibited defects under different stress, we measured mycelial growth by the crossing method but the results indicated that the *Mrmep* mutations did not affect sensitivity to oxidative or osmotic stress (data not shown).

Furthermore, sensitivity of the gene mutations to UV irradiation and thermal stress was examined by measuring conidial germination. We found that conidial germination rates following UV-B irradiation were approximately 51.6, 53.9, and 54.0% for Δ*Mrmep1*, Δ*Mrmep2,* and Δ*Mrmep1*Δ*Mrmep2*, respectively, compared with 47.1% for the WT strain (**Figure 3C**). Similar results were obtained for conidial tolerance to high temperature (40°C); 51.0% of WT conidia germinated, compared with 54.0, 57.2, and 61.3% of conidia for Δ*Mrmep1*, Δ*Mrmep2,* and Δ*Mrmep1*Δ*Mrmep2,* respectively (**Figure 3D**). Through statistical analysis, there was no significant difference in conidial germination between WT and mutant strains after treatment with either UV or heat. Thus, it appears that *Mrmep1* and *Mrmep2* do not play a role in conidial tolerance to either UV irradiation or thermal stress.

### *Mrmep1* and *Mrmep2* Are Required for Full Virulence

**FIGURE 5:**
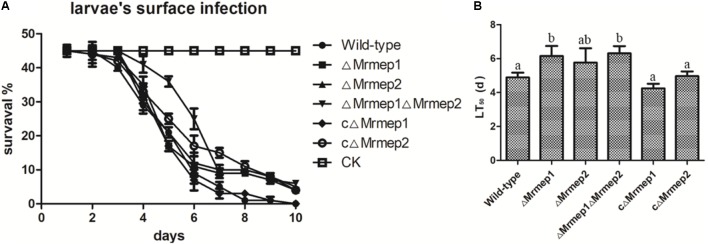
Insect bioassays to assess the loss of *Mrmep1* and *Mrmep2* using the larvae of *G. mellonella* as hosts. **(A)** Survival curves of *G. mellonella* larvae after injection with fungal conidia of WT, Δ*Mrmep1*, Δ*Mrmep2*, Δ*Mrmep1*Δ*Mrmep2*, cΔ*Mrmep1*, or cΔ*Mrmep2* strains. Conidial suspensions (3 μL) at 1 × 10^7^ conidia mL^-1^ were injected into each larva. **(B)** LT_50_ of the different mutant strains.

Insect bioassays using the larvae of *G. mellonella* were employed to assess the consequences of *Mrmep1* and *Mrmep2* loss on fungal virulence. Insects were infected topically—representing the natural route of infection—and mortality was monitored daily over a 12-day period. We found that insects infected with Δ*Mrmep1*, Δ*Mrmep2*, or Δ*Mrmep1*Δ*Mrmep2* displayed an LT_50_ of 6.2, 5.8, and 6.3 days, respectively, while LT_50_ values for WT, cΔ*Mrmep1*, and cΔ*Mrmep2* were 5.0, 4.9, and 4.3 days, respectively. Thus, Δ*Mrmep1*, Δ*Mrmep2*, and Δ*Mrmep1*Δ*Mrmep2* displayed a significant attenuation of virulence (*p* < 0.05) against *G. mellonella* with increased LT_50_ values by 25.5, 19, and 28.8%, respectively, compared with WT (**Figure 5**).

**FIGURE 6:**
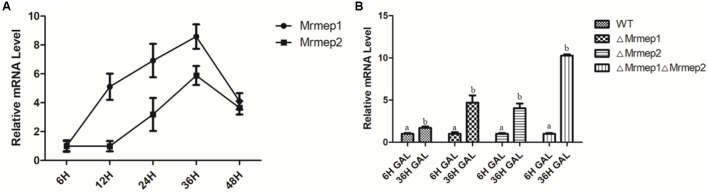
qRT-PCR analysis of the expression levels of different genes in the infection process. **(A)** During *G. mellonella* infection with the WT Mr23, we quantified the relative expression of *Mrmep1* and *Mrmep2* transcript levels in total RNA at 6, 12, 24, 36, and 48 hpi. **(B)** During the infection of *G. mellonella* at 36 hpi, the relative expression levels of the *gal* gene were used to quantify the transcript levels of GAL in the Δ*Mrmep1*, Δ*Mrmep2*, and Δ*Mrmep1*Δ*Mrmep2* strains.

qRT-PCR analysis indicated that expression levels of *Mrmep1* and *Mrmep2* reached a peak at 36 hpi and decreased at 48 hpi, compared with their levels at 6 hpi in the WT Mr23 strain. Furthermore, we also found that the expression levels of *Mrmep1* are always higher than those of *Mrmep2* at each tested time point (**Figure 6A**). To further investigate how insects respond to the fungus, *G. mellonella* individuals were infected with WT, Δ*Mrmep1*, or Δ*Mrmep2* strains and expression levels of the gallerimycin gene were measured. The results showed that GAL mRNA levels were higher in the *Mrmep* mutants than in the WT as they increased by 1.2- and 2.18-fold in the Δ*Mrmep1* and Δ*Mrmep2* mutants, respectively, and 2.5-fold in the Δ*Mrmep1*Δ*Mrmep2* double mutant (**Figure 6B**).

## Discussion

In recent years, a large number of virulence factors have been identified (Wang and Wang, 2017). However, there have been few reports on the biological functions of MEPs in entomopathogenic fungi. Herein, we investigated two Zinc MEPS that are potential virulence factors in *M. robertsii*. Our results suggest that Mrmep1 and Mrmep2 function in growth, sporulation, cell wall integrity, and virulence and that *Mrmep1* is more integral than *Mrmep2* in sporulation.

We found that MrMEP1 and MrMEP2 belong to the ZnMc superfamily based on CDD analysis of their structural domains. Further analysis revealed that MrMEP1 can be subdivided into the ZnMc pappalysin-like family (M43 in the MEROPS database; Laursen et al., 2002) because MrMEP1 includes the characteristic HEVGHWLGLLH motif (where L is a bulky hydrophobic amino acid), coordinates with the zinc ion, and contains a catalytic Glu residue. ZnMc pappalysin-like family has also been linked to pregnancy in humans and other animals (Vallee and Auld, 1990; Tallant et al., 2006). However, it is unclear whether the protein play a similar role in the entomopathogenic fungus. Here, we found that the function of MrMEP1 is related to asexual sporulation in *M. robertsii*.

Disruption of either *Mrmep1* or *Mrmep2* caused an increase in tolerance to CR. However, the double mutant Δ*Mrmep1*Δ*Mrmep2* did not display increased Congo red tolerance in comparison with the single deletion strains. A similar phenomenon was also observed in conidial production. We speculated that the pathway for conidial production or cell wall integrity involving MEPs may have been completely blocked while another pathway was activated for complementation in the Δ*Mrmep1*Δ*Mrmep2* double mutant.

To validate the role of the two MEPs during pathogenesis, we developed *M. robertsii* null mutants lacking the *Mrmep1* or *Mrmep2* genes as well as a double mutant to test their ability in infecting *G. mellonella* larvae and verified the results by qRT-PCR. We found decreased sporulation on the insect cuticle for Δ*Mrmep1* than for Δ*Mrmep2*, which is consistent with the conidial yield of the two mutants.

Moreover, as is well known, gallerimycin is an immune-related gene that confers resistance to fungal pathogens (Altincicek et al., 2007; Huang et al., 2015). Upregulation of gallerimycin at the transcriptional level will increase host defense by the innate immune response (Langen et al., 2006). In our study, the transcription levels of gallerimycin in the single and double mutants were upregulated, suggesting that it was easier for the mutants than for the WT to activate the insect innate immune response.

It has been reported that microbial metalloproteinases mediate the sensing of invading pathogens and activate innate immune responses, including the upregulation of gallerimycin, in *G. mellonella* (Altincicek et al., 2007). Therefore, we speculated that fungal metalloproteinases in *Metarhizium* may also digest hemolymph proteins, which leads to the formation of small peptide fragments that bind to danger-sensing receptors. The engagement of these receptors triggers Toll signaling pathways, resulting in the expression of antimicrobial peptides (AMPs) such as gallerimycin.

Furthermore, our previous research showed that *Mrmep1* and *Mrmep2* expression levels were significantly upregulated in conidia following heat treatment (Wang et al., 2014). Therefore, we speculated that these MEPs may be important in the response to heat stress. However, no apparent phenotypic differences between WT and mutants were observed in the heat stress experiments. This discrepancy between the phenotypic heat stress results and RNA-Seq data may be due to post-transcriptional or post-translational modifications in fungi.

In summary, MrMEP1 and MrMEP2 are Zinc MEPs that function in growth, sporulation, cell wall integrity, and virulence; however, the molecular mechanisms underpinning these functional differences remain unclear. Future studies on downstream signaling pathways could provide further insights.

## Author Contributions

BH, XZ, and ZW conceived and designed the study. RZ wrote the manuscript, conducted the experiments, and analyzed the data. AF performed some of the experiments. BH and ZW edited the manuscript. BH supervised the project. All authors read and approved the final manuscript.

## Conflict of Interest Statement

The authors declare that the research was conducted in the absence of any commercial or financial relationships that could be construed as a potential conflict of interest.

## Footnotes

1 https://www.ncbi.nlm.nih.gov/cdd/

2 http://web.expasy.org/compute_pi/

3 http://www.cbs.dtu.dk/services/SignalP/

4 https://www.ncbi.nlm.nih.gov/

## Supplementary Material

FIGURE S1 Deletion of genes encoding metalloproteases in *M. robertsii*. **(A)** The disruption and complementation plasmids of *Mrmep1*. **(B)** The disruption and complementation plasmids of *Mrmep2*.
