# Supplementary figures and images for: Differential Functions of Two Metalloproteases, Mrmep1 and Mrmep2, in Growth, Sporulation, Cell Wall Integrity, and Virulence in the Filamentous Fungus Metarhizium robertsii
Source: Front Microbiol. 2018 Jul 6;9:1528. doi: 10.3389/fmicb.2018.01528 (PMC6043653; doi:10.3389/fmicb.2018.01528)

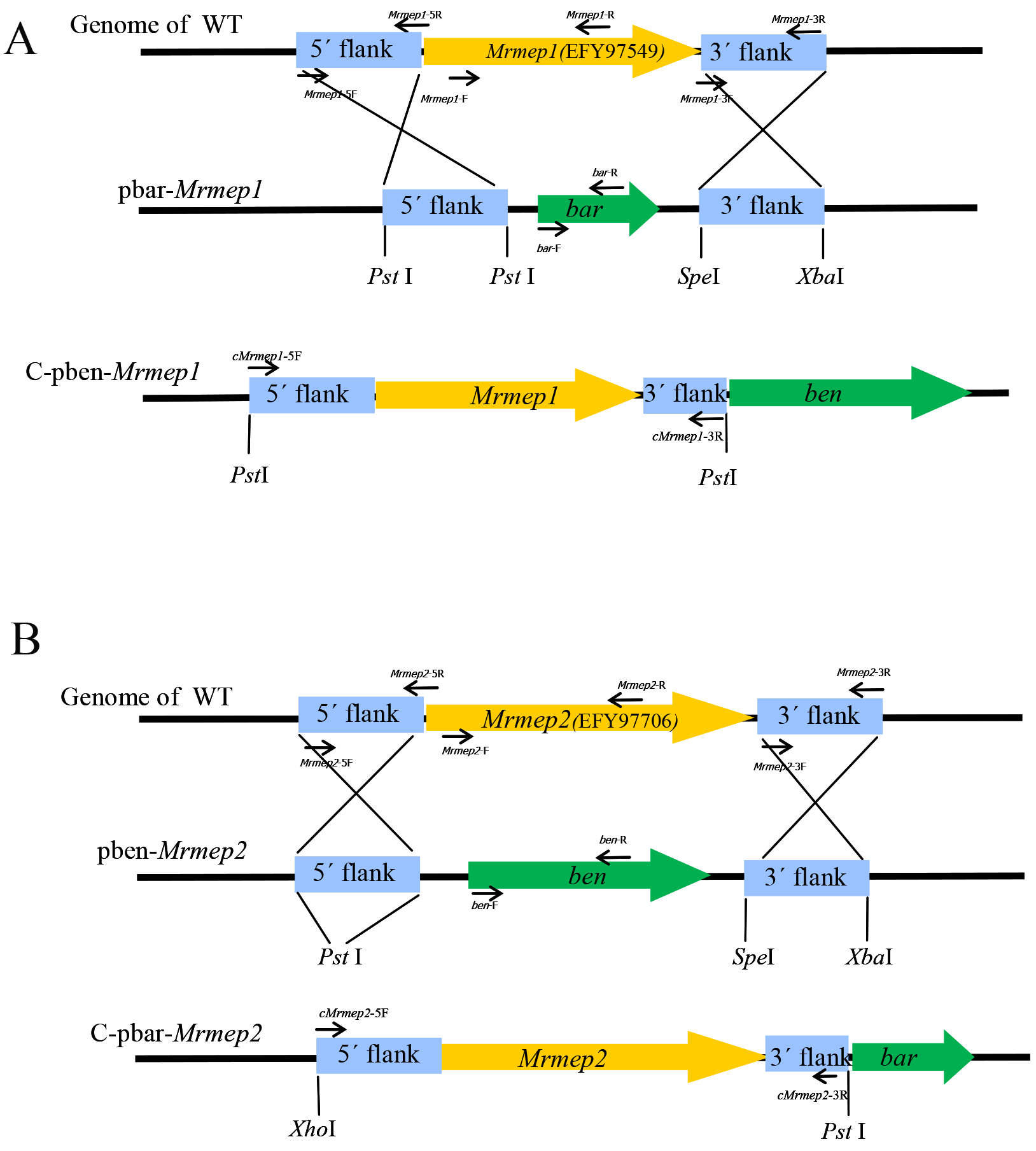

Supplement: FIGURE S1 — Deletion of genes encoding metalloproteases in M. robertsii. (A) The disruption and complementation plasmids of Mrmep1. (B) The disruption and complementation plasmids of Mrmep2. [file Image_1.TIF]
